# Homogenization of multi-institutional chest x-ray images in various data transformation schemes

**DOI:** 10.1117/1.JMI.10.6.061103

**Published:** 2023-04-26

**Authors:** Hyeongseok Kim, Seoyoung Lee, Woo Jung Shim, Min-Seong Choi, Seungryong Cho

**Affiliations:** a KAIST Institute for Artificial Intelligence, Korea Advanced Institute of Science and Technology, Daejeon, Republic of Korea; b Korea Advanced Institute of Science and Technology, Department of Nuclear and Quantum Engineering, Daejeon, Republic of Korea; c AI Research Center, Radisen Co., Ltd., Seoul, Republic of Korea; d KAIST Institute for Health Science and Technology, Korea Advanced Institute of Science and Technology, Daejeon, Republic of Korea; e KAIST Institute for IT Convergence, Korea Advanced Institute of Science and Technology, Daejeon, Republic of Korea

**Keywords:** chest radiograph, deep learning, data transformation, data homogenization, radiomics

## Abstract

**Purpose:**

Although there are several options for improving the generalizability of learned models, a data instance-based approach is desirable when stable data acquisition conditions cannot be guaranteed. Despite the wide use of data transformation methods to reduce data discrepancies between different data domains, detailed analysis for explaining the performance of data transformation methods is lacking.

**Approach:**

This study compares several data transformation methods in the tuberculosis detection task with multi-institutional chest x-ray (CXR) data. Five different data transformations, including normalization, standardization with and without lung masking, and multi-frequency-based (MFB) standardization with and without lung masking were implemented. A tuberculosis detection network was trained using a reference dataset, and the data from six other sites were used for the network performance comparison. To analyze data harmonization performance, we extracted radiomic features and calculated the Mahalanobis distance. We visualized the features with a dimensionality reduction technique. Through similar methods, deep features of the trained networks were also analyzed to examine the models’ responses to the data from various sites.

**Results:**

From various numerical assessments, the MFB standardization with lung masking provided the highest network performance for the non-reference datasets. From the radiomic and deep feature analyses, the features of the multi-site CXRs after MFB with lung masking were found to be well homogenized to the reference data, whereas the others showed limited performance.

**Conclusions:**

Conventional normalization and standardization showed suboptimal performance in minimizing feature differences among various sites. Our study emphasizes the strengths of MFB standardization with lung masking in terms of network performance and feature homogenization.

## 1 Introduction

Learning method estimatations may not generalize well when the joint distribution of inputs and task labels changes substantially.^1^^–^^3^ A network architecture trained on a given domain data set may perform poorly when other domain data are tested. There exist a host of research articles that address this domain discrepancy, and the so-called “domain adaptation” is a subfield of transfer learning^4^^,^^5^ that addresses this problem. A good summary and review of the domain adaptation techniques in chest x-ray (CXR) imaging can be found in the paper of Çallı et. al.^6^ Domain adaptation methods basically seek domain-invariant features or representations between the source and target domains.^7^^–^^11^ These approaches, however, assume that the source domain, from which the samples for testing come, can be appropriately formed and specified. This usually means that enough data from the source domain, specified by a consistent set of scanning hardware, scanning environment, scanning protocol, etc., can be recruited for domain adaptation.^12^^–^^14^

The situation in which we have a particular interest is from the global healthcare disparity perspectives, and it is not supportive for forming such a domain. It is often the case that CXR is performed without satisfying the scanning protocols in medically underserved areas and populations. A lack of enough electric power supply, inadequate data acquisition setting, and absence of licensed technologists are possible causes of inconsistent image quality of CXRs. Considering rather unpredictable scanning conditions, the CXR images at hand may not constitute a well-defined domain in such cases. Artificial intelligence (AI)-enabled techniques are fast evolving in medical fields including automated detection and diagnosis of diseases. They will surely help reducing global healthcare service disparity. It is considered an important area of research and development for such tools to be deployed in the field with their optimal performance uncompromised. The purpose of this study is to implement and compare instance-based data transformation methods in that line of research, which is therefore highly relevant to the special issue of global health, equity, bias, and diversity in AI in medical imaging.

An out-of-distribution detection task that verifies whether a test sample belongs to the predefined source domain can help check the availability of the trained network.^6^^,^^15^ However, to increase the utility of the learned models, a single data instance-based approach, such as input data transformation, is desirable. In this work, we focus on the input transformation approaches and provide a missing link that can explain which method would be more powerful in deep-learning-based detection tasks.

Preprocessing methods are commonly used for deep network performance enhancement^16^ and efficient deep network training.^12^ To reduce data discrepancies, data transformations in the scope of histogram modification techniques, including histogram equalization, matching, clipping, and normalization,^12^^,^^17^ have been investigated. Nevertheless, a claim that such global histogram modification methods cannot harmonize texture differences has been made.^17^ Indeed, data preprocessing steps embracing the multi-frequency characteristics or local features of x-ray images have been reported to improve computer-aided detection^18^^–^^20^ and deep-learning-based detection tasks.^17^^,^^21^^,^^22^ However, it is still questionable whether the results of the network in clinical settings that have different distributions than those used in training can be trusted.^23^^–^^25^ This study compares several data transformation methods in the CXR-based tuberculosis (TB) detection task and provides strong evidence for why one method is superior to the others through radiomics analysis and deep feature analysis.

## 2 Material and Methods

### 2.1 Data Transformation

We implemented three types of transformation algorithms: data normalization, data standardization, and multi-frequency-based (MFB) data standardization. For data standardization and MFB data standardization, we further split each method into two different schemes: with and without lung masks. Therefore, the total number of methods implemented in this work is five. Data normalization and standardization use the transformation Eqs. (1) and (2), respectively.

$$X_{N,i}=\frac{X_{\max\;}^{\mathrm{ref}}-X_{\min}^{\mathrm{ref}}}{X_{\text{max}}-X_{\text{min}}}(X_{i}-X_{\min})+X_{\min}^{\mathrm{ref}},$$ (1)

$$X_{S,i}=\frac{\sigma^{\mathrm{ref}}}{\sigma }(X_{i}-\mu )+\mu^{\mathrm{ref}},$$ (2)

where $X_{i}\in R$ represents a CXR image input pixel value. For an effective normalization of CXR images coming from different systems, a dynamic normalization with histogram analysis is desirable.^12^ Specifically, a DICOM image may contain a constant bias or a letter mark having outlying pixel values. Although some DICOM images provide information that can be utilized to confine pixel value ranges, not all DICOM images provide this information, and windowing parameters are therefore user-specific. In this study, as an attempt to remove such outlying pixels, we first calculated the cumulative histogram f:  R→[0,1] of a given CXR image $X\in R^{N}$. Then, $X_{\min}$ and $X_{\max}$ are such that $f(X_{\min})=0.02$ and $f(X_{\max})=0.98$. In the following homogenization processes, we first applied the above normalization and then calculated image statistics to make images have similar pixel value ranges regardless of the transformation methods. We localized pixels with values that are below $X_{\min}$ or above $X_{\max}$, and those outlier pixels are excluded from the statistics calculation. μ and σ in Eq. (2) indicate the mean and the standard deviation values of the input image after this preprocessing, which are then adjusted to the reference mean $\mu^{\mathrm{ref}}$ and standard deviation $\sigma^{\mathrm{ref}}$. When standardizing images with lung masks, image statistics were calculated only within the masked lung region, whereas the transformation was applied to all of the image pixels.

**Fig. 1:**
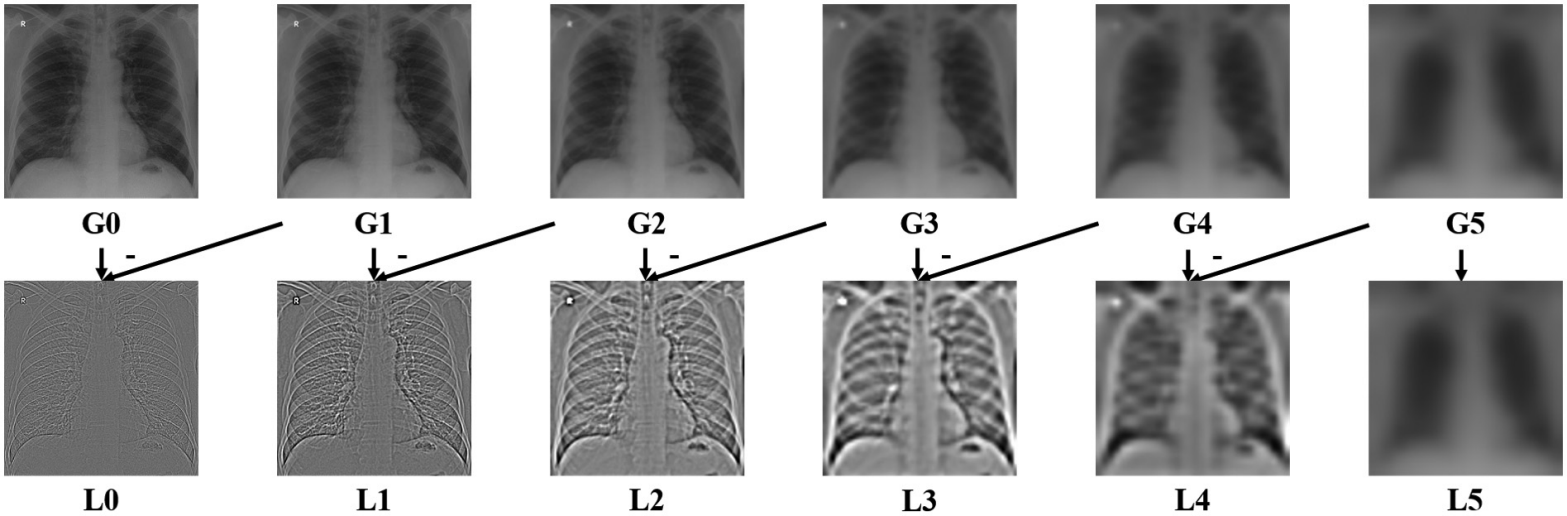
Example Laplacian pyramid of a CXR image.

The MFB data standardization starts with the Laplacian pyramid decomposition,^26^ which is widely used in CXR image enhancement.^27^ The Laplacian pyramid decomposition results in the multiscale representation of a CXR image, and its reverse reconstruction process reproduces the original image. Conventional MFB data enhancement techniques strengthen specific frequency bands of a CXR image, whereas the MFB data standardization aims to make a CXR image similar to the target domain at each multi-frequency band.^18^ Figure 1 shows an example Laplacian pyramid of a CXR image. We specified the Gaussian and the Laplacian pyramid images at the k’th level by $G_{k}$ and $L_{k}$, respectively. Lower frequency information tends to be stored at the higher level of the Laplacian pyramid image by design. For MFB standardization of an input image, we first applied the Laplacian pyramid decomposition to a set of training CXR images. For each training image, the mean $\mu_{k}$ and standard deviation $\sigma_{k}$ values of $L_{k}$ were then calculated. Finally, the reference mean $\mu_{k}^{\mathrm{ref}}$ and standard deviation $\sigma_{k}^{\mathrm{ref}}$ values were calculated by averaging those $\mu_{k}$ and $\sigma_{k}$ values. The MFB standardization process iteratively adjust $\mu_{k}$ and $\sigma_{k}$ values of an input image to $\mu_{k}^{\mathrm{ref}}$ and $\sigma_{k}^{\mathrm{ref}}$ values. As mentioned above, pixels inside the masked lung regions were used for the statistics calculation when the lung masking scheme was adopted. The iterative procedures are summarized in Algorithm 1. We set the total number of iterations N to 50 in this study. We empirically set the maximum level of Laplacian pyramids by 5, which results in a 16×16 array size at the fifth level. The ResNet-18^28^ can downsample the input image with an array size of 512×512 to 16×16, which is equivalent to the minimum array size that is manageable when level 5 is used in the Laplacian pyramid decomposition.

**Algorithm 1:** MFB standardization algorithm

1: **Inputs** X, $\mu_{k}^{\mathrm{ref}}$, $\sigma_{k}^{\mathrm{ref}}$, N
2: $X_{M}^{1}\leftarrow X$
3: **for** i←1 **to** N **do**
4: Decompose $L_{k}^{i}$ from $X_{M}^{i}$
5: Calculate $\mu_{k}^{i}$ and $\sigma_{k}^{i}$ from $L_{k}^{i}\;$
6: ${L^{\prime }}_{k}\leftarrow (L_{k}^{i}-\mu_{k}^{i})\times \frac{\sigma_{k}^{\mathrm{ref}}}{\sigma_{k}^{i}}+\mu_{k}^{\mathrm{ref}}$
7: Reconstruct $X_{M}^{i+1}$ from ${L^{\prime }}_{k}$
8: **end for**
9: **Output** $X_{M}^{N+1}$

### 2.2 Lung Segmentation

To compare the effects on the detection performance of image transformation methods in conjunction with masked lung statistics, we need a lung segmentation tool. Lung segmentation was conducted using a separate deep neural network from the network that is used for TB detection. A network with a Res-UNet structure^29^ was trained with the JSRT CXR dataset^30^ and its mask dataset.^31^ Additionally, we used the China and Montgomery datasets^32^ for additional validation. The training code was implemented using PyTorch libraries^33^ on a system with a GeForce RTX 3090. We summarize the details of the data set used for lung segmentation in Table 1.

**Table 1:** Datasets information for lung segmentation network training.

Site	Number of samples
JSRT (training)	197
JSRT (validation)	50
Montgomery (validation)	138
China (validation)	566

### 2.3 Data Preparation for TB Detection

We used multi-institutional CXR data from seven clinical sites to train and test the deep neural network for TB detection. Anonymized datasets were collected from the clinically cooperative institutions of the Radisen AI Research Center. This study was approved by the institutional review boards, and informed consent was waived. We summarize dataset information in Table 2. It should be noted that the reference site data provide the target domain examples and other sites provide the source domain examples and that the source domains are diverse in terms of CXR modality, bit-depths, and scanning protocols, of which details are unavailable. The reference dataset is composed of half normal CXR and half abnormal CXR diagnosed with TB. For non-reference data, there are class imbalances between TB sample sizes and normal sample sizes. Because data skewness affects performance metrics,^34^ we calculated the performance metrics after under-sampling normal data, so the sample sizes of the two classes become equal. The under-sampling was randomly performed and repeated, so statistical analyses are feasible. Meanwhile, we used all of the data for the feature analyses because the class imbalance itself does not have a signification influence on the feature extraction.

**Table 2:** Multi-institutional datasets information.

Site	Modality	Data bits	Number of normal samples	Number of TB samples
Reference (training)	DR	16	956	956
Reference (test)	DR	16	250	250
A (test)	CR	16	250	77
B (test)	CR	14	248	41
C (test)	DR	12	244	136
D (test)	CR	12	285	40
E (test)	CR	12	247	37
F (test)	CR	10	297	26

### 2.4 TB Detection Network Training

**Fig. 2:**
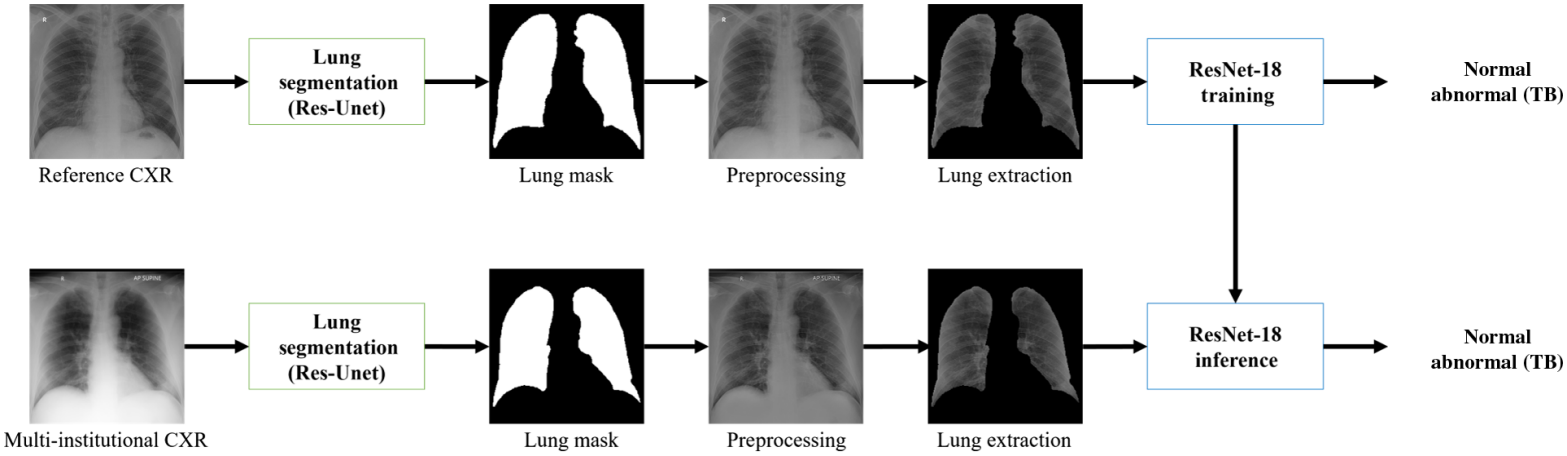
Diagram of overall procedures for comparison study.

The overall workflow of the CXR-based TB detection in this work is shown in Fig. 2. In the training phase, lung regions were first identified using the trained lung segmentation network. After appropriate image cropping, each image was downsized to an array of 512×512 using bilinear interpolation. Various data transformation methods were then applied to each image and then the lung regions of the processed images were used for the network training. In Fig. 2, we omitted the image cropping process for the simplicity of presentation. ResNet-18 architecture was used for the TB detection network. Training details are presented in Appendix A.

### 2.5 TB Detection Performance Evaluation

In the inference phase, CXR images from seven different sites in Table 2 were tested. For testing each TB detection network trained by the data that went through a specific data transformation, the same data transformation method was applied to the input CXR image. For example, we applied the MFB data standardization with lung masking to the test dataset when we evaluated the performance of a TB detection network that was trained by the data transformed by the MFB standardization with lung masking. For the evaluation, receiver operating characteristic (ROC) and precision-recall (PR) curves were used. We also calculated an F1 score of the reference test results with 20 different threshold values within [0, 1]. The threshold value that provides the highest F1 score in the reference test results was applied to other datasets to calculate F1 scores and recalls. For the reference dataset, we bootstrapped the performance metrics for statistical analysis. Random bootstrapping was performed up to a thousand times, which was determined so that the standard deviation from the bootstrapping results stay within 2% of difference from the Delong’s estimated standard deviation in the area under the curve (AUC) value of ROC curves.^35^^,^^36^ For non-reference data (from sites A to F), we randomly repeated the under-sampling a thousand times, which is the same number of times that bootstrapping was performed.

Localization of suspicious regions in the CXR image was also performed using Grad-Cam++,^37^ which can provide the activation map for TB detection. The bounding boxes were drawn by radiologists for suspicious areas in the entire data set, and we evaluated how well the obtained saliency maps match the radiologists’ insights. We achieved Grad-Cam++ images at the deepest feature layer and calculated the weighted intersection over attention (WIOA) values with respect to the radiologists’ bounding box information. Grad-CAM++ images and WIOA values were produced from the M3d-CAM PyTorch library.^38^ Details on the procedure for calculating the WIOA can be found in Appendix B.

### 2.6 Radiomic Features

To observe the distributions of various datasets, we first extracted the radiomic features^39^^–^^42^ of CXR images transformed by the aforementioned methods. For each processed CXR image, we masked out the region outside of the lung. PyRadiomics^43^ was used to extract features from the preprocessed lung images. We calculated 93 radiomic features, which can be grouped into 6 different series including: first-order statistics, gray-level co-occurrence matrix (GLCM), gray-level dependence matrix (GLDM), gray-level run-length matrix, gray-level size zone matrix, and neighboring gray tone difference matrix. The last five features can be grouped into second-order statistics. We did not include shape features because the shape of the lung in the CXR is not the target of data homogenization.

We calculated the Mahalanobis distance^44^^,^^45^ as a numerical assessment of the homogenization ability of the radiomic features. The Mahalanobis distance $D_{M}$ is a distance between a point x and a distribution D with a mean of $\vec{\mu }$ and a covariance matrix S and is defined as

$$D_{M}(\overset{\to }{x})=\sqrt{{\left(\overset{\to }{x}-\overset{\to }{\mu }\right)}^{T}S^{-1}(\overset{\to }{x}-\overset{\to }{\mu })}.$$ (3)

We calculated the distances between each sample of test sites (A to F) and the distribution of reference data. The distances of first- and second-order radiomics were calculated separately.

For a better visualization of the features, we performed a principal component analysis (PCA) of the calculated radiomics. We chose PCA for visualization to compare the effects of data harmonization on radiomic features in the common coordinate system. Because the radiomic features of images were extracted in the same way regardless of the dataset and harmonization methods, such a comparison is legitimate.

### 2.7 Deep Features

The architecture of a typical convolutional neural network (CNN)^46^^,^^47^ consists of a series of convolutional layers and pooling layers. We focused on the final layer of the CNN because it stores all of the essential information extracted from the input image in the form of a feature vector. In this work, we used the latent feature vector of the final layer of the TB detection network (Sec. 2.4), which is a vector with a size of 512. We used t-distributed stochastic neighbor embedding (t-SNE)^48^ to visualize the latent feature vector. The t-SNE helps with understanding how the network responds to the test data under various data transformation through visualizing the feature clusters.

## 3 Results

### 3.1 Data Transformation

**Fig. 3:**
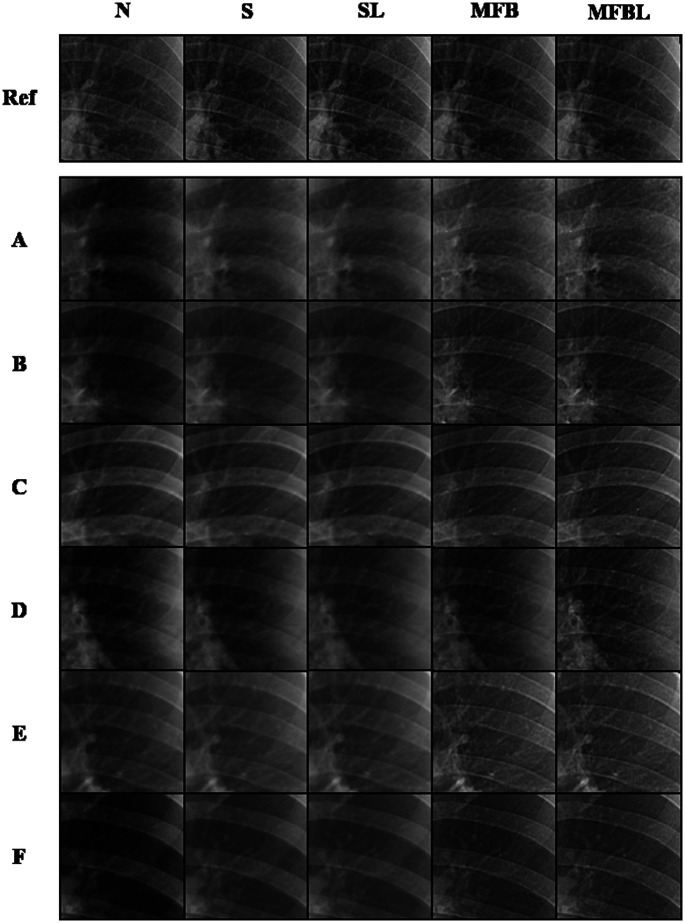
Patches extracted from inputs to TB detection networks with different data transformations. Images in the same column are displayed with the same display window.

Figure 3 shows the example network inputs of lung-segmented images processed by different transformation methods from various sites. N, S, SL, MFB, and MFBL indicate data normalization, data standardization without and with lung masking, MFB data standardization without and with lung masking, respectively. In Fig. 3, the data N method resulted in non-uniform lung region brightness. It is observed that MFB methods generally provide a more similar appearance overall with the reference compared with the N, S and SL in the same display window setting. N, S and SL resulted in rather coarse image textures in other site datasets compared with those in the reference, whereas MFB methods produced finer textures similar to the reference. However, the MFB without a lung masking scheme provides suboptimal visual texture similarity in some data, for example, site D in Fig. 3. We included a full breakdown of the time spent on various preprocessing techniques in Appendix C.

### 3.2 TB Detection Performance

**Fig. 4:**
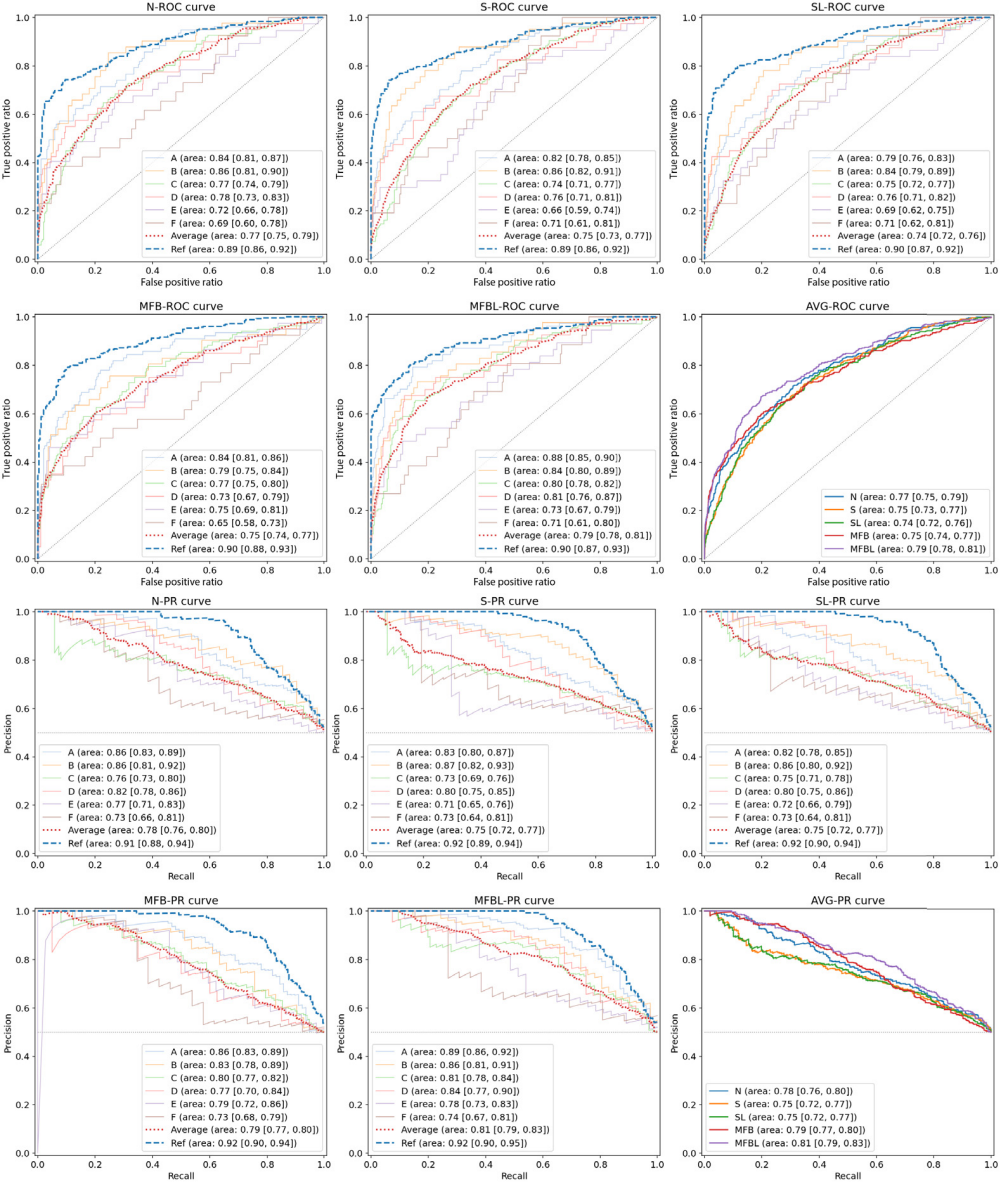
ROC and PR curves with different data transformations. Each line shows the ROC or PR curve for each site. The area value in the legend means the AUC and AP values for ROC and PR curves, respectively. Values in square brackets represent the 95% confidence interval. Average lines were produced by collecting every result from site A to site F.

In Fig. 4, we show the ROC and the PR curves of the network outputs from different data transformation methods. The AUC values of the ROC and the average precision (AP) values of the PR curves for the reference data showed marginal network performance differences among various data transformations. Network performances, however, largely varied for the six other site datasets. The MFBL method resulted in the minimum gap between the reference curve and the site-average curve in both ROC and PR, whereas S and SL showed poorer results in both AUC and AP.

**Fig. 5:**
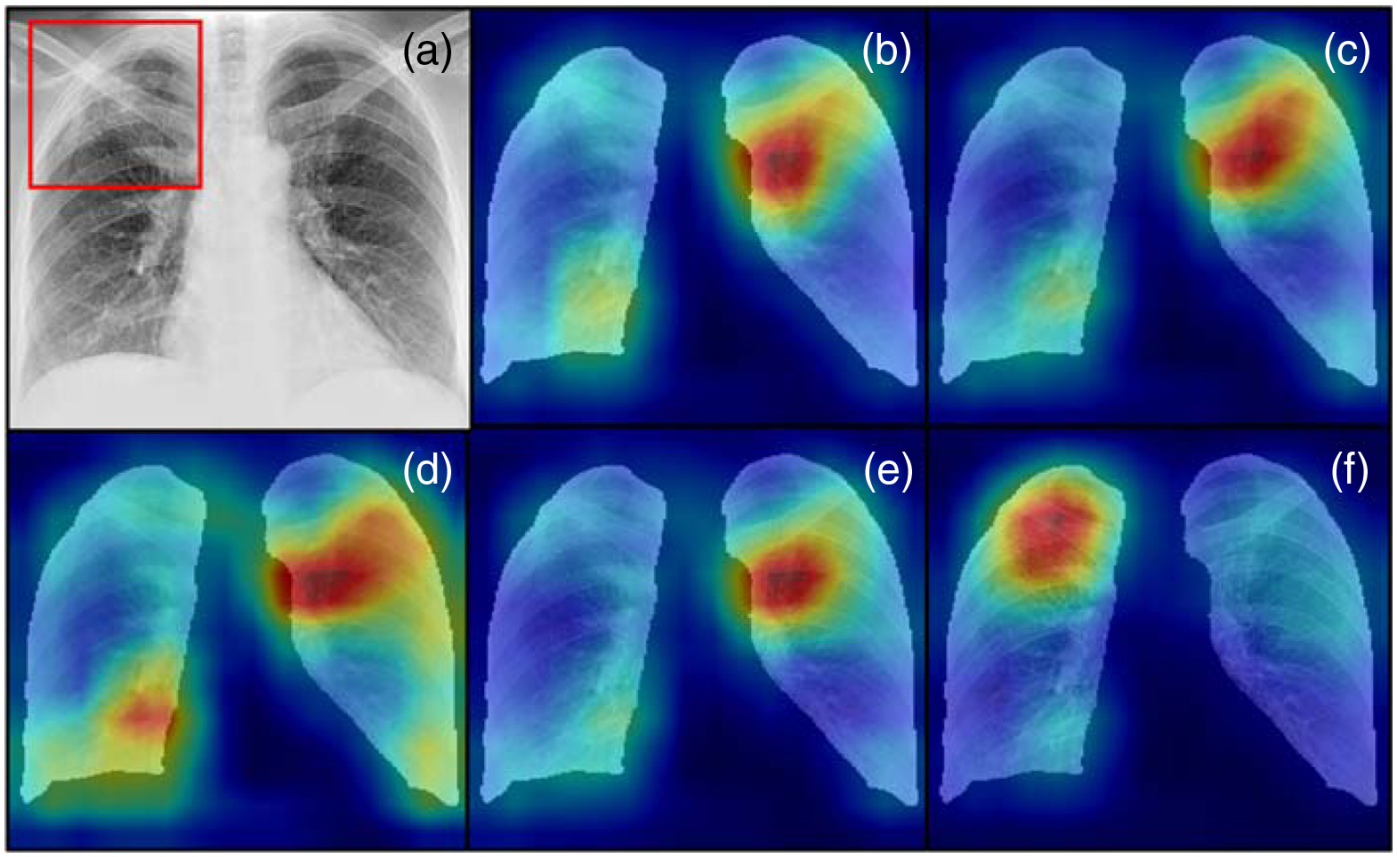
(a) Bounding box generated by radiologist and example GCam++ images of set D with different data transformations, (b) N, (c) S, (d) SL, (e) MFB, and (f) MFBL.

Table 3 summarizes the network performance evaluation results over the sites in terms of F1 score, recall, and WIOA. Here, please note that the average values in the table are average scores from sites A to F, and we present the standard deviation of those six scores. In the F1 score result, the MFBL method showed the highest values for every non-reference site except F. It is noted that site variation of the scores is minimum in the MFB and MFBL results, which implies more robustness of the network performance. In the recall score, average recall scores of the N, S, and SL methods did not exceed 0.5, which means they failed to detect true TB cases at a >50% chance. Considering that we balanced the classes by undersampling, the chance would be lower than random calls. The WIOA value of a non-trained network specified as random in Table 3 was around 0.31. The WIOA values of the reference dataset with different networks were around 0.60, and the MFBL methods provided slightly lower values in the non-reference datasets as well. Figure 5 shows examples of bounding boxes and Grad-CAM++ images. As shown in Fig. 5(f), a hot spot in the saliency map from the MFBL network goes well with the radiologist’s bounding box.

**Table 3:** Network quantitative evaluation summary.

		Ref	A	B	C	D	E	F	Average
F1	N	0.80	0.62	0.55	0.17	0.46	0.59	0.21	0.42 ± 0.20
	S	0.82	0.62	0.57	0.22	0.14	0.63	0.32	0.45 ± 0.17
	SL	0.83	0.45	0.50	0.22	0.10	0.61	0.27	0.36 ± 0.18
	MFB	0.83	0.77	0.71	0.51	0.56	0.65	0.56	0.63 ± 0.09
	MFBL	0.81	0.78	0.75	0.70	0.75	0.68	0.54	0.72 ± 0.09
Recall	N	0.75	0.47	0.39	0.10	0.30	0.49	0.12	0.27 ± 0.17
	S	0.77	0.49	0.41	0.13	0.25	0.62	0.19	0.31 ± 0.19
	SL	0.78	0.30	0.34	0.13	0.07	0.59	0.15	0.23 ± 0.19
	MFB	0.80	0.75	0.66	0.36	0.50	0.62	0.46	0.53 ± 0.14
	MFBL	0.77	0.87	0.73	0.62	0.75	0.76	0.46	0.70 ± 0.14
WIOA	N	0.61	0.54	0.51	0.35	0.37	0.53	0.41	0.45 ± 0.08
	S	0.59	0.49	0.42	0.36	0.35	0.50	0.38	0.42 ± 0.07
	SL	0.60	0.45	0.40	0.33	0.33	0.49	0.35	0.39 ± 0.07
	MFB	0.59	0.57	0.51	0.39	0.48	0.58	0.48	0.50 ± 0.07
	MFBL	0.62	0.61	0.57	0.48	0.52	0.62	0.54	0.56 ± 0.06
	*Random	0.39	0.32	0.33	0.24	0.30	0.41	0.28	0.31 ± 0.06

### 3.3 Radiomic Features

**Fig. 6:**
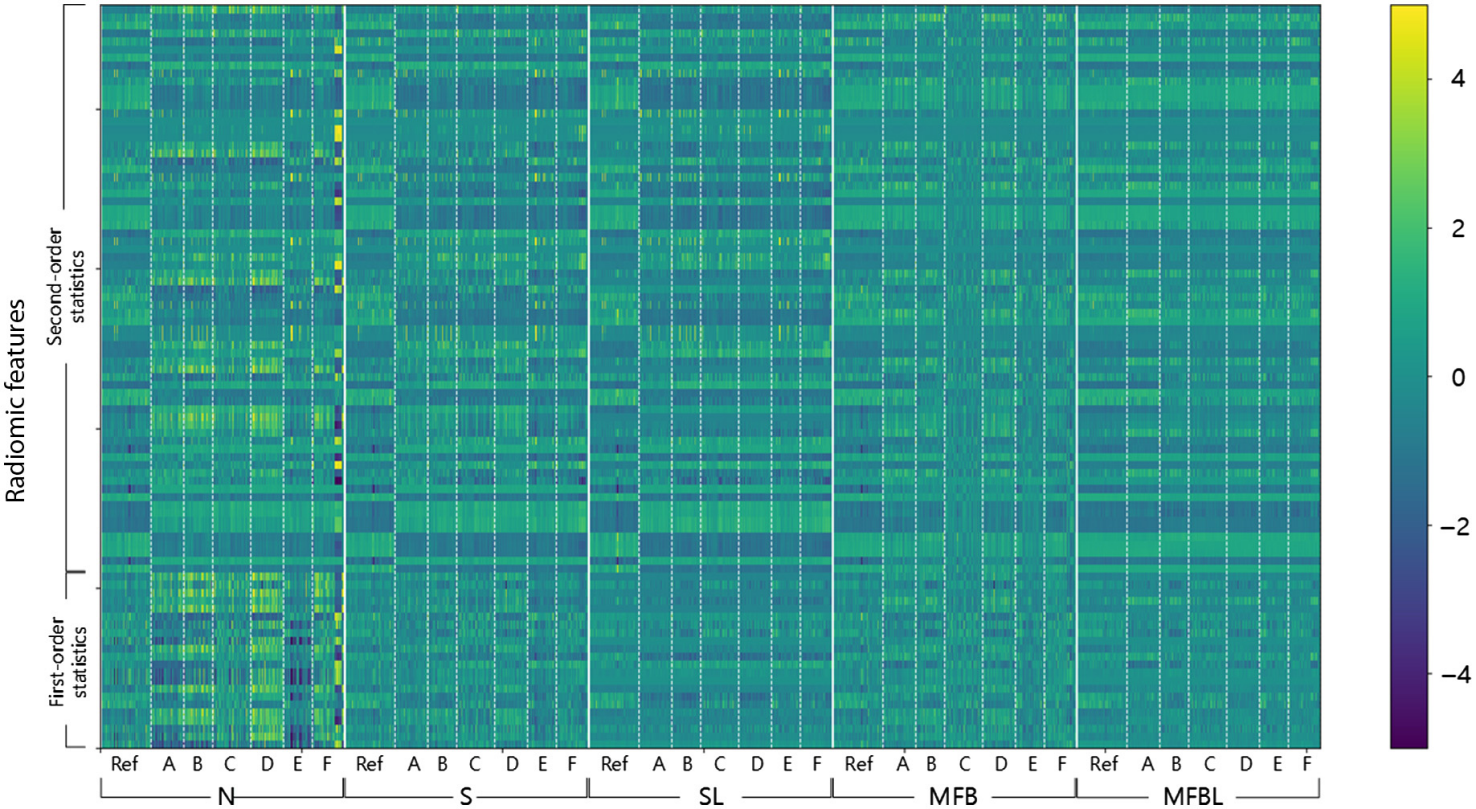
Heatmap depicting z-scores of 93 radiomic features for various datasets with a conventional N and four different homogenization methods. The vertical axis denotes radiomic features, divided into first-order statistics and second-order statistics including GLCM, and GLDM. The horizontal axis denotes samples. Note that the MFBL method showed the best homogenization results throughout various features.

For a visual comparison, we calculated the Z-scores of the features and presented a heatmap of the Z-scores. Figure 6 shows the corresponding heatmap, with each row representing one feature and each column representing one CXR image. The heatmap is divided into five sections on the horizontal axis; each section denotes each data transformation method. If Z-scores of a certain radiomic feature are similar across the samples from different sites, it can be said that the homogenization performance of the method is proper in terms of that specific radiomic feature. The N cannot harmonize data from the multi-site data. S and SL, worked as intended for the first-order statistics. However, there was a clear discrepancy between reference test data and other multi-site data in terms of higher-order radiomic features. MFB succeeds in reducing the discrepancies for various sites both in first- and second-order radiomic features. The use of lung masks seems to be more effective in terms of harmonization performance. The heatmap suggests that the MFBL can harmonize not only the histogram characteristic but also textural features.

**Fig. 7:**
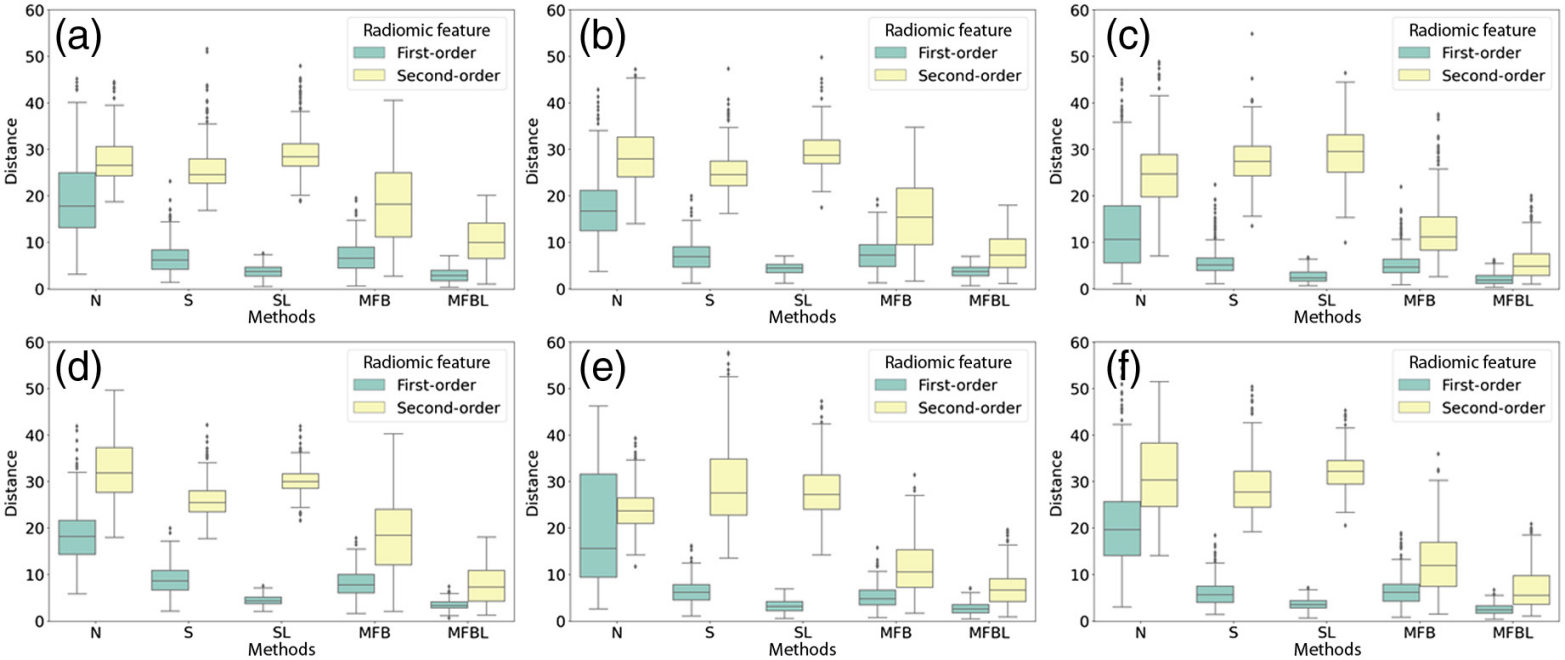
Box-and-whisker plots of the Mahalanobis distances between test radiomic features and the reference radiomic features. (a)–(f) The site A, B, C, D, E, and F. N, S, and SL cannot harmonize the second-order statistics well, whereas the performance of the MFBL was the best for all sites.

Figure 7 is a box-and-whisker plot of Malalanobis distances between the features of test and reference sites. A larger distance implies that a corresponding feature is not well harmonized, albeit after a certain data transformation method, and vice versa. The implications of the box plots of distances are in the same vein as the results shown qualitatively in the heatmap (Fig. 6). The N has a limited performance on feature harmonization overall. S and SL can harmonize only first-order statistics and not second-order features. MFBL resulted in the shortest feature distances; implying a superior homogenization performance.

Note that the smaller discrepancy in radiomics feature space does not always guarantee better performance of the classification network. For example, although the S improved the first-order feature in all cases compared with N, the performance of the network is generally better for the normalized images (see Fig. 4). Nevertheless, we confirmed that conventional N and S failed to harmonize the textural features of the images as compared to the MFB and thus cannot substantially reduce the effect of reference data bias.

**Fig. 8:**
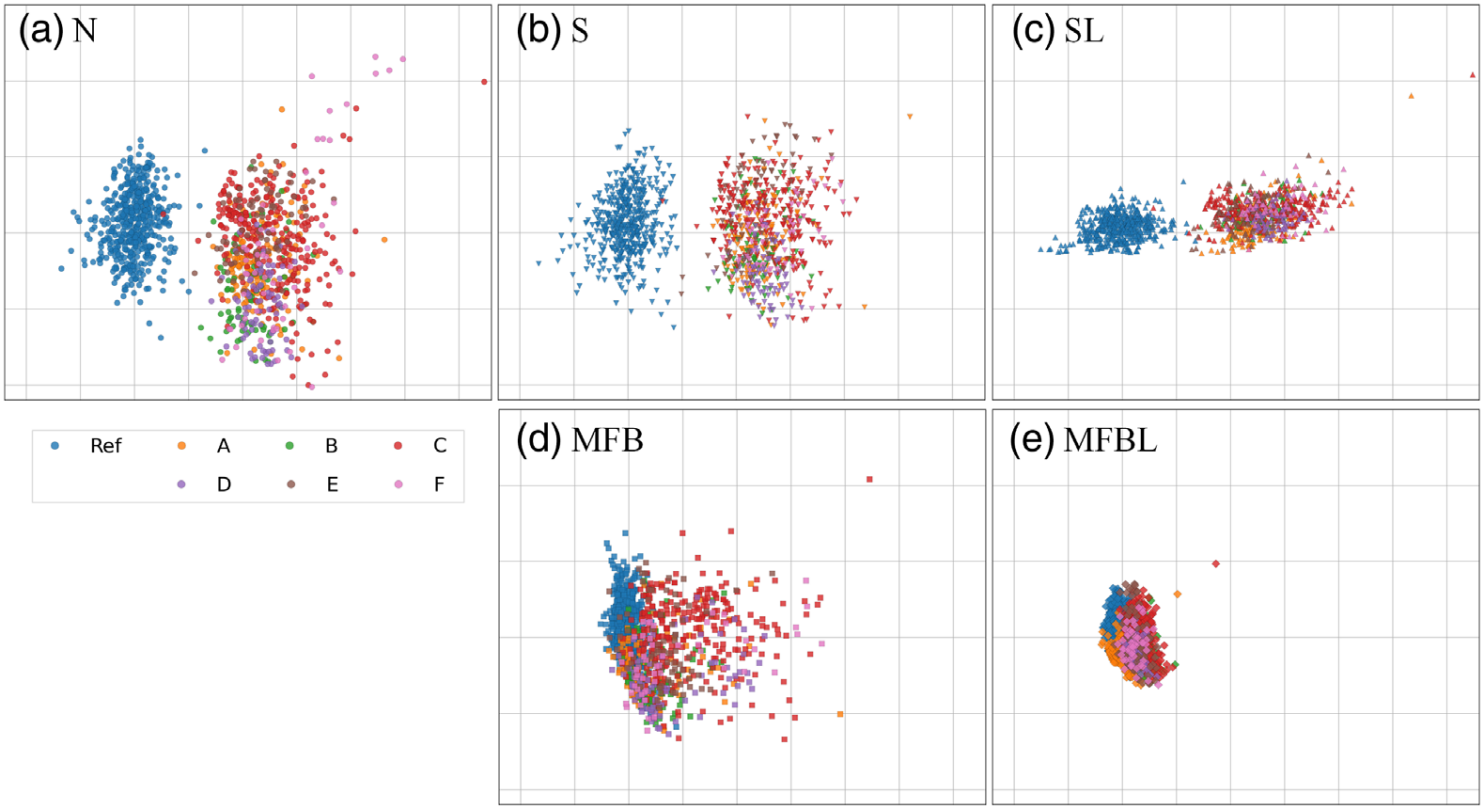
Visualizations of radiomic features after data transformation. (a) N, (b) S, (c) SL, (d) MFB, and (e) MFBL. Visualizations are done by PCA of high-dimensional radiomic feature vectors.

Figure 8 contains visualizations of radiomic features after five different data transformation processes. There are distinct differences between the radiomic features of the reference site and other sites that have undergone conventional N and S. However, after MFB, especially with lung masks [Fig. 8(e)], it is clear that the radiomic features of the samples from different sites are well harmonized with the reference group.

### 3.4 Deep Features

**Fig. 9:**
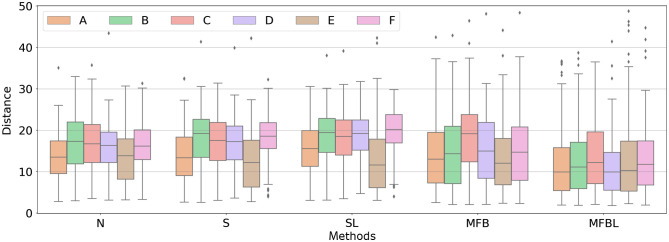
Box-and-whisker plot of the Mahalanobis distance of the deep features. Distances were calculated between each point in a dataset to the reference distribution.

Table 4 summarizes a list of the calculated Mahalanobis distances of the multi-site features to the reference distribution used for the network training. Figure 9 shows the box-and-whisker plots corresponding to Table 4. The MFBL method shows the closest distance regardless of the test data. This implies that the deep feature harmonization ability of the MFBL method is the highest and other harmonization methods may have failed to match the data distributions. Similar to the result of radiomic feature analysis, the distance in the deep feature space is not necessarily inversely correlated to the network classification performance. However, the distance can be used as a metric to determine the network’s trustworthiness for data having different distributions from the training data.

**Table 4:** Mahalanobis distances ($D_{M}$) for multi-site data homogenization. The target distribution was the reference train dataset. A larger distance indicates an outlier to the target distribution.

	A	B	C	D	E	F
N	13.55 ± 5.458	16.82 ± 6.530	16.86 ± 6.473	15.62 ± 5.640	13.73 ± 6.248	16.77 ± 5.109
S	13.62 ± 6.123	17.86 ± 6.655	17.33 ± 6.526	16.85 ± 5.810	12.80 ± 7.181	18.44 ± 5.157
SL	15.38 ± 5.939	18.37 ± 6.315	18.17 ± 6.666	18.63 ± 5.039	12.83 ± 7.769	19.98 ± 4.810
MFB	13.93 ± 7.837	14.83 ± 8.461	18.20 ± 8.367	15.46 ± 8.028	13.46 ± 7.749	14.67 ± 7.791
MFBL	11.66 ± 7.681	12.54 ± 7.789	13.76 ± 8.294	10.72 ± 6.124	12.54 ± 8.831	12.55 ± 7.010

**Fig. 10:**
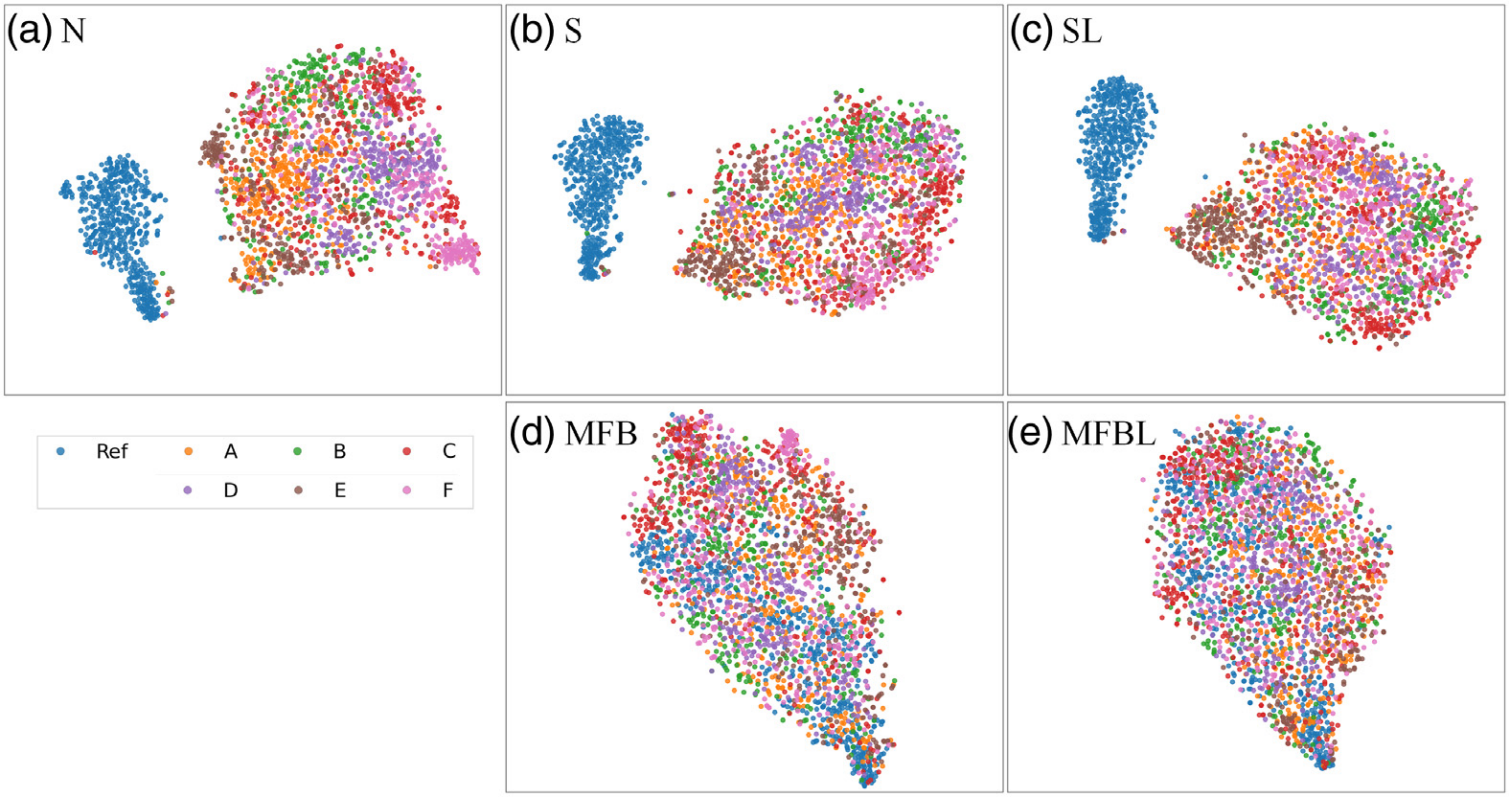
t-SNE visualizations of deep-embedded features after conventional N and four different data transformations. (a) N, (b) S, (c) SL, (d) MFB, and (e) MFBL. Note that, for N and S, the deep features are not well harmonized.

The t-SNE visualization results are presented in Fig. 10. After N, S, and SL [Fig. 10(a)–10(c)], the intrasite differences were reduced, but the homogenization between reference data and other sites was not successful; multi-site data forms a distinct cluster from the reference. From radiomic feature analysis (Sec. 3.3), we found that N, S and SL could not reduce data distribution between reference and other sites. The t-SNE result implies that the network was not able to handle the reference data bias and finally resulted in a biased model. From the point of view of the TB detection network learned from the reference dataset, it can be seen that data from other sites are still treated as out-of-distribution. Although the network may have achieved a somewhat satisfying performance after N, S or SL (Sec. 3.2), the latent vector analysis does not provide strong support for such an improvement. Meanwhile, both versions of MFB methods can harmonize multi-site data in terms of the network’s deep feature, not forming any distinct cluster [Figs. 10(d) and 10(e)].

## 4 Discussion

In this study, we used a single dataset from a single clinical site for training and tried to harmonize other datasets with the reference data exploiting data transformation methods. Using multi-institutional datasets for training in conjunction with data augmentation is also a viable option for a network’s generalizability enhancement. This approach can be interpreted as one that increases the diversity of feature space of the training dataset, hopefully covering the feature space of a new dataset. Pixel-level transformations, including contrast or brightness adjustment and spatial-level transformation, such as shift and rotation, were used in an example work.^13^ Our study shows the importance of image features at multiscale representations in the detection network training. Developing data augmentation strategies with image frequency modulation is planned as our future study.

It is observed that radiomic and deep features from the multi-site dataset after the N, S, or SL method did not agree well with the features of the reference test data. However, it is shown repeatedly that the features within the multi-sites become similar to each other after N, S and SL [see Figs. 8(a)–10(c), Figs. 10(a)–10(c)]. It is perhaps due to the fact that the textures of test data from A to F are similar in the original images. As shown in Fig. 4, the reference image is rather sharp and accordingly high-frequency-emphasized, whereas the images of other sites after N, S and SL are blurrier in a similar fashion.

Deep learning has achieved great success in various image processing tasks, and domain adaptation is one of the benefited applications. Unsupervised deep-neural-network-based domain adaption methods aim to transform the data distribution of the source domain to the target domain.^7^^,^^10^^,^^49^ By such a domain transformation, data harmonization between chest radiographs acquired from various conditions can be achieved. However, there are two shortcomings with such translation methods, which can be major obstacles, especially in medically underserved regions.

One reason is that deep learning techniques require a considerable amount of data. Recent studies, motivated by generative models, utilize the generative adversarial networks (GAN)^50^-based model to generate domain-matched data from the target to the source.^51^^–^^54^ The performance of generative models heavily depends on the number of images for training, and the data-efficient models still require hundreds of images.^55^

Another point is that deep neural networks are computationally heavy. The problem remains for the few-shot learning techniques because the CNNs have a large number of parameters to be trained. The computational burden of training the network in a new environment inevitably becomes an obstacle to rapid diagnosis. If the chest x-ray scanner is placed on a mobile system, it is impractical to mount such a high-performance computing device to the system.

**Fig. 11:**
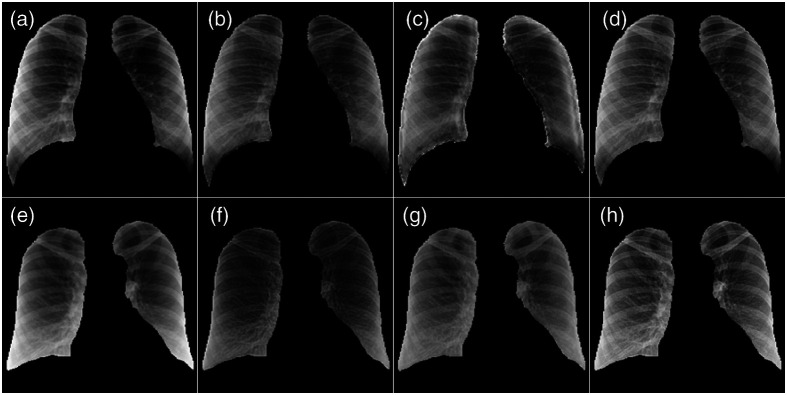
Comparison of the deep-learning-based one-shot domain translation and the MFBL transformation. Each image corresponds to (a, e) normalized data from dataset A, (b, f) outputs of a network trained on (a), (c, g) outputs of a network trained on (e), and (d, h) MFBL.

There are alternative versions of GAN such as one- or few-shot domain transfer models that require smaller datasets to train. Because we are interested in instance-based data harmonization, we implemented a one-shot GAN learning model.^56^ A network was trained to align the domain features of dataset A to the reference data. Lung-masked images were used for training. The process was accelerated by a single NVIDIA GeForce GTX 1080 Ti graphics processing unit, and it took two days to train a single one-shot domain translation network. Two distinct networks were trained on two different samples of dataset A, and generative-model-based domain translation resulted in significantly varying outcomes, depending on which instance was used for one-shot training (Please see Fig. 11). However, the transformations in this paper’s scope are fully instance-based and thus free from such complications.

In this study, we analyzed the data features after various harmonizations. For the quantitative analysis, we calculated the Mahalanobis distance between the deep features of reference data and the test data points. The conclusion was that the MFBL, which resulted in the shortest distance, is the most efficient harmonization. Unfortunately, it was difficult to establish the direct correlation between the distance in feature space and the degree of harmonization. For example, the average deep feature distance of N (13.73) is farther than that of S (12.80) in the case of dataset E (see Table 4), whereas the network performance in terms of AUC and AP was superior for the N case. Still, there is an overall tendency that the shorter the distance is, the better the network performance metric becomes. Although further research is needed to determine the criteria for a reliable and credible model that is less affected by the reference data distribution, our suggested feature analysis may provide a basis for such discussion.

We would like to emphasize that this study contributes to understanding the data homogenization processes through feature analyses and recommends the MFBL method as the instance-based data transformation method potentially for the CXR images acquired at medically underserved areas. As the special issue of global health, equity, bias, and diversity in AI in medical imaging is pursued, this contribution will help to reduce global healthcare disparity and diversity.

## 5 Conclusions

In this work, we implemented various instance-based data transformations to reduce data discrepancy for the multi-institutional use of a trained deep-learning prediction model. A CNN-based TB detection network was trained using the reference site data, and the TB detection performance was tested for the remaining six sites after applying N, S, SL, MFB, and MFBL. MFBL outperformed other methods in terms of numerical criteria including AUC, AP, F1 score, and WIOA. For the radiomic feature analysis, we calculated the Mahalanobis distance and performed dimensionality reduction. We found that S and SL match the histogram-based features well but fail to match the texture-related second-order statistics. On the other hand, the textural features of the multi-site CXRs after MFBL were well homogenized to the reference data. The deep features of the trained network were analyzed through the same method, and the MFBL showed the best harmonization performance. Conventional N and S, on the other hand, did not lessen the distribution gap between multi-site datasets and were accordingly unsuccessful in deep feature harmonization. Our study emphasizes the strengths of the MFBL, especially its comparative advantage on network performance and ability to lessen the disparity between various data distributions.

## 6 Appendix A: TB Detection Network Training Details

We initialized every model corresponding to each data transformation method with the pretrained ResNet-18 provided by PyTorch, and the same random seed was used for training. A dropout layer having a dropout ratio of 0.6 was added before the last linear layer. We used the same computing resources that were used for the lung segmentation network training, and training details are summarized in Table 5. The early stopping condition was determined by monitoring the validation losses with the reference test dataset.

**Table 5:** TB detection network training details.

Task	TB detection
Loss function	BCE with logits loss
Optimizer	SGD with momentum
Learning scheduler	Cosine annealing warm restart^57^
Maximum learning rate	$1e^{-4}$
Batch size	128
Number of total epochs	1200

## 7 Appendix B: Calculation of WIOA

**Fig. 12:**
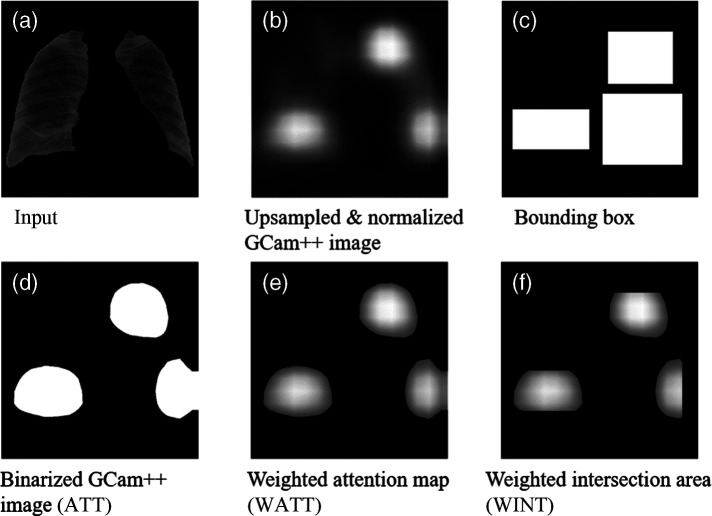
(a) Example input image to explain WIOA value calculation, (b) the GCam++ image of the corresponding input image, achieved at the deepest feature layer, (c) the bounding box of TB regions drawn by a radiologist, (d) the binarized GCam++ image with thresholding, (e) the weighted attention map, (b) multiplied with (d), (f) the weighted intersection area, and (e) multiplied with (c). The WIOA value was defined as the summation of the pixel values of (f) divided by that of (e).

In Fig. 12, we show the procedure for calculating the WIOA value in an exemplary case. The Grad-Cam++ image is first segmented by the Otsu method,^58^ which results in a binarized attention map ATT. Then, a weighted attention map WATT is generated by multiplying the Grad-Cam++ image and ATT. The multiplication of the WATT and the bounding boxes provides the weighted intersection area WINT, and the WIOA value is finally calculated by

$$\mathrm{WIOA}=\frac{\sum_{x}\mathrm{WINT}\;(x)\;}{\sum_{x}\mathrm{WATT}(x)}.$$ (4)

## 8 Appendix C: Data Preprocessing Time

Table 6 provides a detailed analysis of the time required for each data-transforming procedure. It is important to mention that we preprocessed the entire dataset prior to the actual training and saved the results separately. Through this strategy, we could avoid increasing the training time. Specifically, the preprocessing of ∼2000 reference data required an additional 4 minutes to complete.

**Table 6:** Data preprocessing time. All measurements were done in intel Xeon® CPU E3-1270v5/GTX 1080 Ti platform.

Preprocessing	Approximate time (s)
Dicom read	0.004
N	0.28
Lung segmentation	0.04
Downsampling processes	0.25
S	0.004
MFB	0.13

As is summarized above, in addition to the reference computation time (0.28 s), only a few 100 milliseconds are additionally required for the lung segmentation and MFB. We believe this increase in computation time would not hamper its use in clinical practices.

## 9 Appendix D: Learning Curve

**Fig. 13:**
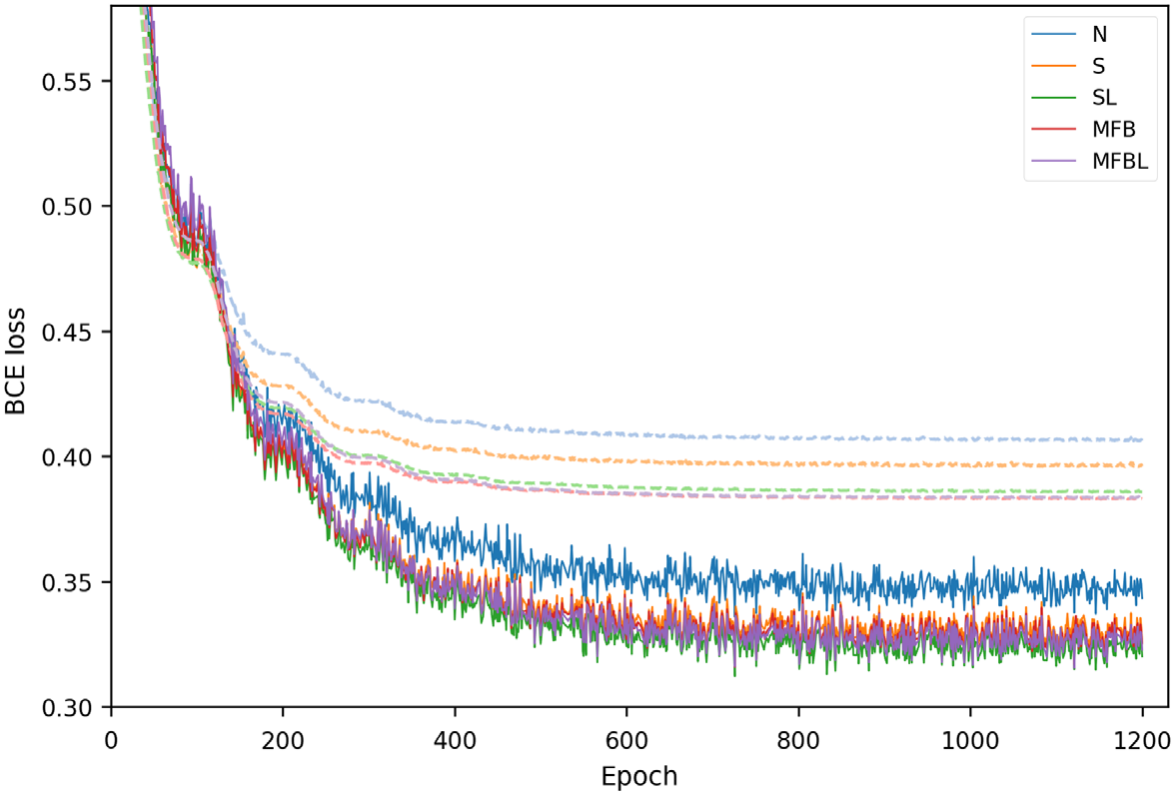
Training and validation loss plots of the networks.

In terms of convergence in the training phase, all of the implemented harmonization methods showed a similar speed of convergence, as shown in Fig. 13. The training phase used 1200 epochs in all cases. Solid lines and dashed lines represent the training phase and the validation phase, respectively.

## References

[1] Samala R. K. et al., “Generalization error analysis for deep convolutional neural network with transfer learning in breast cancer diagnosis,” Phys. Med. Biol. 65(10), 105002 (2020). PHMBA7 0031-9155 10.1088/1361-6560/ab82e8 32208369 PMC7981191

[r2] El Naqa I. M. et al., “Lessons learned in transitioning to AI in the medical imaging of COVID-19,” J. Med. Imaging 8(S1), 010902 (2021). JMEIET 0920-5497 10.1117/1.JMI.8.S1.010902 PMC8488974 34646912

[3] Cohen J. P. et al., “On the limits of cross-domain generalization in automated X-ray prediction,” in Proc. Third Conf. Med. Imaging with Deep Learn. 121, pp. 136–155 (2020).

[4] Chaddad A. Hassan L. Desrosiers C., “Deep CNN models for predicting COVID-19 in CT and x-ray images,” J. Med. Imaging 8(S1), 014502 (2021). JMEIET 0920-5497 10.1117/1.JMI.8.S1.014502 PMC8071782 33912622

[5] Manokaran J. et al., “Detection of COVID-19 from chest x-ray images using transfer learning,” J. Med. Imaging 8(S1), 017503 (2021). JMEIET 0920-5497 10.1117/1.JMI.8.S1.017503 PMC8382139 34435075

[6] Çallı E. et al., “Deep learning for chest X-ray analysis: a survey,” Med. Image Anal. 72, 102125 (2021). 10.1016/j.media.2021.102125 34171622

[7] Zhao H. et al., “On learning invariant representations for domain adaptation,” in 36th Int. Conf. Mach. Learn. ICML 2019, pp. 12985–12999 (2019).

[r8] Wang R. Chaudhari P. Davatzikos C., “Embracing the disharmony in medical imaging: a simple and effective framework for domain adaptation,” Med. Image Anal. 76, 102309 (2022). 10.1016/j.media.2021.102309 34871931 PMC8792340

[r9] Pan S. J. et al., “Domain adaptation via transfer component analysis,” IEEE Trans. Neural Networks 22(2), 199–210 (2011). ITNNEP 1045-9227 10.1109/TNN.2010.2091281 21095864

[r10] Ganin Y. Lempitsky V., “Unsupervised domain adaptation by backpropagation,” in 32nd Int. Conf. Mach. Learn. ICML 2015, Vol. 2 (2015).

[11] Jiang J. et al., “Self-derived organ attention for unpaired CT-MRI deep domain adaptation based MRI segmentation,” Phys. Med. Biol. 65(20), 205001 (2020). PHMBA7 0031-9155 10.1088/1361-6560/ab9fca 33027063

[12] Gündel S. et al., “Robust classification from noisy labels: integrating additional knowledge for chest radiography abnormality assessment,” Med. Image Anal. 72, 102087 (2021). 10.1016/j.media.2021.102087 34015595

[r13] Tang Y. X. et al., “Automated abnormality classification of chest radiographs using deep convolutional neural networks,” NPJ Digital Med. 3(1), 70 (2020). 10.1038/s41746-020-0273-z PMC7224391 32435698

[14] Zheng J. et al., “Pairwise domain adaptation module for CNN-based 2-D/3-D registration,” J. Med. Imaging 5(2), 021204 (2018). JMEIET 0920-5497 10.1117/1.JMI.5.2.021204 PMC5767648 29376104

[15] Márquez-Neila P. Sznitman R., “Image data validation for medical systems,” Lect. Notes Comput. Sci. 11767, 329–337 (2019). LNCSD9 0302-9743 10.1007/978-3-030-32251-9_36

[16] Singh D. Singh B., “Investigating the impact of data normalization on classification performance,” Appl. Soft Comput. 97, 105524 (2020). 10.1016/j.asoc.2019.105524

[17] Vidya M. S. et al., “Local and global transformations to improve learning of medical images applied to chest radiographs,” Proc. SPIE 10949, 1094936 (2019). PSISDG 0277-786X 10.1117/12.2512717

[18] Philipsen R. H. H. M. et al., “Localized energy-based normalization of medical images: application to chest radiography,” IEEE Trans. Med. Imaging 34(9), 1965–1975 (2015). ITMID4 0278-0062 10.1109/TMI.2015.2418031 25838517

[r19] Philipsen R. H. H. M. et al., “Automated chest X-ray reading for tuberculosis in the Philippines to improve case detection: a cohort study,” Int. J. Tuberc. Lung Dis. 23(7), 805–810 (2019). 10.5588/ijtld.18.0004 31439111

[20] Murphy K. et al., “Computer aided detection of tuberculosis on chest radiographs: an evaluation of the CAD4TB v6 system,” Sci. Rep. 10, 5492 (2020). SRCEC3 2045-2322 10.1038/s41598-020-62148-y 32218458 PMC7099074

[21] De Moor T. et al., “Automated lesion detection and segmentation in digital mammography using a u-net deep learning network,” Proc. SPIE 10718, 1071805 (2018). PSISDG 0277-786X 10.1117/12.2318326

[22] Murphy K. et al., “COVID-19 on chest radiographs: a multireader evaluation of an artificial intelligence system,” Radiology 296(3), E166–E172 (2020). RADLAX 0033-8419 10.1148/radiol.2020201874 32384019 PMC7437494

[23] Wu J. T. Y. et al., “Developing and validating multi-modal models for mortality prediction in COVID-19 patients: a multi-center retrospective study,” J. Digital Imaging 35, 1514–1529 (2022). JDIMEW 10.1007/s10278-022-00674-z PMC9255527 35789446

[r24] Roberts M. et al., “Common pitfalls and recommendations for using machine learning to detect and prognosticate for COVID-19 using chest radiographs and CT scans,” Nat. Mach. Intell. 3(3), 199–217 (2021). 10.1038/s42256-021-00307-0

[25] DeGrave A. J. Janizek J. D. Lee S. I., “AI for radiographic COVID-19 detection selects shortcuts over signal,” Nat. Mach. Intell. 3(7), 610–619 (2021). 10.1038/s42256-021-00338-7

[26] Burt P. J. Adelson E. H., “The Laplacian pyramid as a compact image code,” Readings Comput. Vision 671–679 (1987). 10.1016/B978-0-08-051581-6.50065-9

[27] Vuylsteke P. Schoeters E. P., “Multiscale image contrast amplification (MUSICA),” Proc. SPIE 2167, 551–560 (1994). PSISDG 0277-786X 10.1117/12.175090

[28] He K. et al., “Deep residual learning for image recognition,” in Proc. IEEE Comput. Soc. Conf. Comput. Vision Pattern Recognit., pp. 770–778 (2016). 10.1109/CVPR.2016.90

[29] Huang X. et al., “Metal artifact reduction on cervical CT images by deep residual learning,” Biomed. Eng. Online 17(1), 175 (2018). 10.1186/s12938-018-0609-y 30482231 PMC6260559

[30] Shiraishi J. et al., “Development of a digital image database for chest radiographs with and without a lung nodule: receiver operating characteristic analysis of radiologists’ detection of pulmonary nodules,” Am. J. Roentgenol. 174(1), 71–74 (2000). AJROAM 0092-5381 10.2214/ajr.174.1.1740071 10628457

[31] van Ginneken B. Stegmann M. B. Loog M., “Segmentation of anatomical structures in chest radiographs using supervised methods: a comparative study on a public database,” Med. Image Anal. 10(1), 19–40 (2006). 10.1016/j.media.2005.02.002 15919232

[32] Jaeger S. et al., “Two public chest X-ray datasets for computer-aided screening of pulmonary diseases,” Quantum Imaging Med. Surg. 4(6), 475–477 (2014). 10.3978/j.issn.2223-4292.2014.11.20 PMC4256233 25525580

[33] Paszke A. et al., “Automatic differentiation in pytorch,” in NIPS 2017 Work. Autodiff. (2017).

[34] Williams C. K. I., “The effect of class imbalance on precision-recall curves,” Neural Comput. 33(4) (2021). NEUCEB 0899-7667 10.1162/neco_a_01362 33513323

[35] DeLong E. R. DeLong D. M. Clarke-Pearson D. L., “Comparing the areas under two or more correlated receiver operating characteristic curves: a nonparametric approach,” Biometrics 44(3), 837–845 (1988). BIOMB6 0006-341X 10.2307/2531595 3203132

[36] Sun X. Xu W., “Fast implementation of DeLong’s algorithm for comparing the areas under correlated receiver operating characteristic curves,” IEEE Signal Process. Lett. 21(11), 1389–1393 (2014). IESPEJ 1070-9908 10.1109/LSP.2014.2337313

[37] Chattopadhay A. et al., “Grad-CAM++: generalized gradient-based visual explanations for deep convolutional networks,” in Proc. - 2018 IEEE Winter Conf. Appl. Comput. Vision, pp. 839–847 (2018). 10.1109/WACV.2018.00097

[38] Gotkowski K. et al., “M3d-CAM: a PyTorch library to generate 3D data attention maps for medical deep learning,” in Bildverarbeitung für die Medizin pp. 217–222 (2021). 10.1007/978-3-658-33198-6_52

[39] Gillies R. J. Kinahan P. E. Hricak H., “Radiomics: images are more than pictures, they are data,” Radiology 278(2), 563–577 (2016). RADLAX 0033-8419 10.1148/radiol.2015151169 26579733 PMC4734157

[r40] Rizzo S. et al., “Radiomics: the facts and the challenges of image analysis,” Eur. Radiol. Exp. 2(1), 36 (2018). 10.1186/s41747-018-0068-z 30426318 PMC6234198

[r41] Yip S. S. F. Aerts H. J. W. L., “Applications and limitations of radiomics,” Phys. Med. Biol. 61(13), R150–R166 (2016). PHMBA7 0031-9155 10.1088/0031-9155/61/13/R150 27269645 PMC4927328

[42] Kontos D. Summers R. M. Giger M. L., “Special section guest editorial: Radiomics and deep learning,” J. Med. Imaging 4(4), 041301 (2018). JMEIET 0920-5497 10.1117/1.JMI.4.4.041301 PMC5752704 29322066

[43] van Griethuysen J. J. M. et al., “Computational radiomics system to decode the radiographic phenotype,” Cancer Res. 77(21), e104–e107 (2017). CNREA8 0008-5472 10.1158/0008-5472.CAN-17-0339 29092951 PMC5672828

[44] De Maesschalck R. Jouan-Rimbaud D. Massart D. L., “The Mahalanobis distance,” Chemom. Intell. Lab. Syst. 50(1), 1–18 (2000). 10.1016/S0169-7439(99)00047-7

[45] Lee K. et al., “A simple unified framework for detecting out-of-distribution samples and adversarial attacks,” in Adv. Neural Inf. Process. Syst., pp. 7167–7177 (2018).

[46] Schmidhuber J., “Deep learning in neural networks: an overview,” Neural Networks 61, 85–117 (2015). NNETEB 0893-6080 10.1016/j.neunet.2014.09.003 25462637

[47] LeCun Y. Bengio Y. Hinton G., “Deep learning,” Nature 521(7553), 436–444 (2015). 10.1038/nature14539 26017442

[48] van Der Maaten L. Hinton G., “Visualizing data using t-SNE,” J. Mach. Learn. Res. 9, 2579–2605 (2008).

[49] Guo S. et al., “Multi-level semantic adaptation for few-shot segmentation on cardiac image sequences,” Med. Image Anal. 73, 102170 (2021). 10.1016/j.media.2021.102170 34380105

[50] Goodfellow I. J. et al., “Generative adversarial nets,” in Adv. Neural Inf. Process. Syst., Vol. 3(Jan.) (2014).

[51] Liu M. Y. Tuzel O., “Coupled generative adversarial networks,” in Adv. Neural Inf. Process. Syst. (2016).

[r52] Hoffman J. et al., “CyCADA: cycle-consistent adversarial domain adaptation,” in 35th Int. Conf. Mach. Learn., Vol. 5 (2018).

[r53] Tzeng E. et al., “Adversarial discriminative domain adaptation,” in Proc. - 30th IEEE Conf. Comput. Vis. Pattern Recognit. (2017). 10.1109/CVPR.2017.316

[54] Huang S. W. et al., “AugGAN: cross domain adaptation with GAN-based data augmentation,” Lect. Notes Comput. Sci. 11213 731–744 (2018). LNCSD9 0302-9743 10.1007/978-3-030-01240-3_44

[55] Zhao S. et al., “Differentiable augmentation for data-efficient GAN training,” in Adv. Neural Inf. Process. Syst. (2020).

[56] Benaim S. Wolf L., “One-shot unsupervised cross domain translation,” in Adv. Neural Inf. Process. Syst., pp. 2104–2114 (2018).

[57] Loshchilov I. Hutter F., “SGDR: stochastic gradient descent with warm restarts,” in 5th Int. Conf. Learn. Represent. ICLR 2017 - Conf. Track Proc. (2017).

[58] Otsu N., “A threshold selection method from gray-level histograms,” IEEE Trans Syst Man Cybern 9(1), 62–66 (1979). 10.1109/TSMC.1979.4310076

